# On almost *B*-summable double sequence spaces

**DOI:** 10.1186/s13660-017-1606-6

**Published:** 2018-01-08

**Authors:** Orhan Tuğ

**Affiliations:** Department of Mathematics Education, Ishik University, 100 meter street, Erbil, Iraq

**Keywords:** 46A45, 40C05, four-dimensional matrices, $B(r,s,t,u)$-summable, four-dimensional matrix domain, almost convergent double sequence space, *α*-, $\beta(\vartheta)$- and *γ*-dual, four-dimensional matrix mapping

## Abstract

The concept of a four-dimensional generalized difference matrix and its domain on some double sequence spaces was recently introduced and studied by Tuğ and Başar (AIP Conference Proceedings, vol. 1759, 2016) and Tuğ (J. Inequal. Appl. 2017(1):149, 2017). In this present paper, as a natural continuation of (J. Inequal. Appl. 2017(1):149, 2017), we introduce new almost null and almost convergent double sequence spaces $B(\mathcal{C}_{f})$ and $B(\mathcal{C}_{f_{0}})$ as the four-dimensional generalized difference matrix $B(r,s,t,u)$ domain in the spaces $\mathcal{C}_{f}$ and $\mathcal{C}_{f_{0}}$, respectively. Firstly, we prove that the spaces $B(\mathcal{C}_{f})$ and $B(\mathcal{C}_{f_{0}})$ of double sequences are Banach spaces under some certain conditions. Then we give an inclusion relation of these new almost convergent double sequence spaces. Moreover, we identify the *α*-dual, $\beta(bp)$-dual and *γ*-dual of the space $B(\mathcal{C}_{f})$. Finally, we characterize some new matrix classes $(B(\mathcal{M}_{u}):\mathcal{C}_{f})$, $(\mathcal{M}_{u}:B(\mathcal {C}_{f}))$, and we complete this work with some significant results.

## Preliminaries, background and notation

We denote the set of all complex valued double sequence by Ω, which is a vector space with coordinatewise addition and scalar multiplication. Any subspace of Ω is called a double sequence space. A double sequence $x=(x_{mn})$ of complex numbers is called bounded if $\Vert x \Vert _{\infty}=\sup_{m,n\in\mathbb{N}} \vert x_{mn} \vert <\infty $, where $\mathbb{N}=\{0,1,2,\ldots\}$. The space of all bounded double sequences is denoted by $\mathcal{M}_{u}$, which is a Banach space with the norm $\Vert \cdot \Vert _{\infty}$. Consider the double sequence $x=(x_{mn})\in\Omega$. If for every $\epsilon>0$ there exist a natural number $n_{0}=n_{0}(\epsilon)$ and $l\in\mathbb{C}$ such that $\vert x_{mn}-l \vert <\epsilon$ for all $m,n>n_{0}$, then the double sequence *x* is said to be convergent in Pringsheim’s sense to the limit point *l* says that $p-\lim_{m,n\rightarrow\infty}x_{mn}=l$; where $\mathbb{C}$ indicates the complex field. The space $\mathcal{C}_{p}$ denotes the set of all convergent double sequences in Pringsheim’s sense. Although every convergent single sequence is bounded, this is not hold for double sequences in general. That is, there are such double sequences which are convergent in Pringsheim’s sense but not bounded. Actually, Boos [3, p. 16] defined the sequence $x=(x_{mn})$ by

$$\begin{aligned} x_{mn}= \textstyle\begin{cases} n& m=0, n\in\mathbb{N}; \\ 0& m\geq1, n\in\mathbb{N}. \end{cases}\displaystyle \end{aligned}$$

Then it is clearly seen that $p-\lim_{m,n\rightarrow\infty}x_{mn}=0$ but $\Vert x \Vert _{\infty}=\sup_{m,n\in\mathbb{N}} \vert x_{mn} \vert =\infty$, so $x\in\mathcal{C}_{p}-\mathcal{M}_{u} $. Now, we may denote the space of all both convergent in Pringsheim’s sense and bounded double sequences by the set $\mathcal{C}_{bp}$, *i.e.*, $\mathcal {C}_{bp}=\mathcal{C}_{p}\cap\mathcal{M}_{u}$. Hardy [4] showed that a double sequence $x=(x_{mn})$ is said to converge regularly to *l* if $x\in\mathcal{C}_{p}$ and the limits $x_{m}:=\lim_{n} x_{mn}, (m\in\mathbb{N})$ and $x_{n}:=\lim_{m} x_{mn}, (n\in\mathbb {N})$ exist, and the limits $\lim_{m}\lim_{n} x_{mn}$ and $\lim_{n}\lim_{m} x_{mn}$ exist and are equal to the *p*-limit of *x*. Moreover, by $\mathcal{C}_{bp0}$ and $\mathcal{C}_{r0}$, we may denote the spaces of all null double sequences contained in the sequence spaces $\mathcal {C}_{bp}$ and $\mathcal{C}_{r}$, respectively. Móricz [5] proved that the double sequence spaces $\mathcal{C}_{bp}$, $\mathcal {C}_{bp0}$, $\mathcal{C}_{r}$ and $\mathcal{C}_{r0}$ are Banach spaces with the norm $\Vert \cdot \Vert _{\infty}$. The space $\mathcal{L}_{q}$ of all absolutely *q*-summable double sequences corresponding to the space $\ell_{q}$ of *q*-summable single sequences was defined by Başar and Sever [6], that is,

$$\begin{aligned} \mathcal{L}_{q}:= \biggl\{ x=(x_{kl})\in\Omega:\sum _{k,l} \vert x_{kl} \vert ^{q}< \infty \biggr\} \quad (1\leq q< \infty), \end{aligned}$$

which is a Banach space with the norm $\Vert \cdot \Vert _{q}$. Then the space $\mathcal{L}_{u}$, which is a special case of the space $\mathcal{L}_{q}$ with $q=1$, was introduced by Zeltser [7].

Let *λ* be a double sequence space and converging with respect to some linear convergence rule is $\vartheta-\lim:\lambda\to \mathbb{C}$. Then the sum of a double series $\sum_{i,j}x_{ij}$ relating to this rule is defined by $\vartheta-\sum_{i,j}x_{ij}=\vartheta-\lim_{m,n\to\infty}\sum_{i,j=0}^{m,n}x_{ij}$. Throughout, the summation from 0 to ∞ without limits, that is, $\sum_{i,j}x_{ij}$ means that $\sum_{i,j=0}^{\infty}x_{ij}$.

Here and below, unless stated otherwise we consider that *ϑ* denotes any of the symbols $p,bp$ or *r*.

The *α*-dual $\lambda^{\alpha}$, the $\beta(\vartheta)$-dual $\lambda^{\beta(\vartheta)}$ with respect to the *ϑ*-convergence and the *γ*-dual $\lambda^{\gamma}$ of the double sequence space *λ* are, respectively, defined by

$$\begin{aligned} &\lambda^{\alpha}:= \biggl\{ a=(a_{kl})\in\Omega:\sum _{k,l} \vert a_{kl}x_{kl} \vert < \infty \text{ for all } x=(x_{kl})\in\lambda \biggr\} , \\ &\lambda^{\beta(\vartheta)}:= \biggl\{ a=(a_{kl})\in\Omega :\vartheta-\sum _{k,l}a_{kl}x_{kl} \text{ exists for all } x=(x_{kl})\in\lambda \biggr\} , \\ &\lambda^{\gamma}:= \Biggl\{ a=(a_{kl})\in\Omega:\sup _{m,n\in \mathbb{N}} \Biggl\vert \sum_{k,l=0}^{m,n}a_{kl}x_{kl} \Biggr\vert < \infty \text{ for all } x=(x_{kl})\in\lambda \Biggr\} . \end{aligned}$$

It is easy to see for any two spaces *λ* and *μ* of double sequences that $\mu^{\alpha}\subset\lambda^{\alpha}$ whenever $\lambda\subset\mu$ and $\lambda^{\alpha}\subset\lambda^{\gamma }$. Additionally, it is well known that the inclusion $\lambda^{\alpha }\subset\lambda^{\beta(\vartheta)}$ holds, while the inclusion $\lambda^{\beta(\vartheta)}\subset\lambda^{\gamma}$ does not hold, since the *ϑ*-convergence of the double sequence of partial sum of a double series does not guarantee its boundedness.

Here, we shall be concerned with a four-dimensional matrix transformation from any double sequence space *λ* to any double sequence space *μ*. Given any four-dimensional infinite matrix $A=(a_{mnkl})$, where $m,n,k,l\in\mathbb{N}$, any double sequence $x=(x_{kl})$, we write $Ax=\{(Ax)_{mn}\}_{m,n\in\mathbb{N}}$ for the *A*-transform of *x*, exists for every sequence $x=(x_{kl})\in\lambda $ and it is in *μ*; here

1.1 $$\begin{aligned} (Ax)_{mn}=\vartheta-\sum_{k,l}a_{mnkl}x_{kl}\quad \text{for each } m,n\in\mathbb{N}. \end{aligned}$$

The four-dimensional matrix domain has fundamental importance for this article. Therefore, this concept is presented in this paragraph. The *ϑ*-summability domain $\lambda_{A}^{(\vartheta)}$ of *A* in a space *λ* of double sequences is described as

$$\begin{aligned} \lambda_{A}^{(\vartheta)}= \biggl\{ x=(x_{kl})\in\Omega: Ax= \biggl(\vartheta-\sum_{k,l}a_{mnkl}x_{kl} \biggr)_{m,n\in\mathbb {N}}\text{ exists and is in }\lambda \biggr\} . \end{aligned}$$

The notation (1.1) says that *A* maps the space *λ* into the space *μ* if $\lambda\subset\mu_{A}^{(\vartheta)}$ and we denote the set of all four-dimensional matrices, transforming the space *λ* into the space *μ*, by $(\lambda:\mu)$. Thus, $A=(a_{mnkl})\in(\lambda:\mu)$ if and only if the double series on the right side of (1.1) converges in the sense of *ϑ* for each $m,n\in\mathbb{N}$, *i.e*., $A_{mn}\in\lambda ^{\beta(\vartheta)}$ for all $m,n\in\mathbb{N}$ and we have $Ax\in \mu$ for all $x\in\lambda$; where $A_{mn}=(a_{mnkl})_{k,l\in\mathbb {N}}$ for all $m,n\in\mathbb{N}$. Moreover, the following definitions are significant in order to classify the four-dimensional matrices. A four-dimensional matrix *A* is called $\mathcal{C}_{\vartheta }$-conservative if $\mathcal{C}_{\vartheta}\subset(\mathcal {C}_{\vartheta})_{A}$, and it is called $\mathcal{C}_{\vartheta }$-regular if it is $\mathcal{C}_{\vartheta}$-conservative and

$$\begin{aligned} \vartheta-\lim Ax=\vartheta-\lim_{m,n\to\infty}(Ax)_{mn}= \vartheta -\lim_{m,n\to\infty}x_{mn}, \quad\text{where } x=(x_{mn})\in\mathcal {C}_{\vartheta}. \end{aligned}$$

By using the notations of Zelster [8] we may define the double sequences $e^{kl}=(e_{mn}^{kl}), e^{1}, e_{k}$ and *e* by

$$\begin{aligned} &e_{mn}^{kl}= \textstyle\begin{cases} 1 &(k,l)=(m,n); \\ 0 & \mbox{otherwise}, \end{cases}\displaystyle \\ &e^{1}=\sum_{k}e^{kl},\qquad e_{k}=\sum_{l}e^{kl} \quad\text{and}\quad e=\sum_{kl}e^{kl} \end{aligned}$$

for all $k,l,m,n\in\mathbb{N}$ and we may write the set Φ by $\Phi=\operatorname{span}\{e^{kl}: k,l\in\mathbb{N}\}$.

In order to establish a new sequence space, special triangular matrices were previously used. These new spaces derived by the domain of matrices are expansions or contractions of the original space, in general. Adams [9] called the four-dimensional infinite matrix $A=(a_{mnkl})$ a triangular matrix if $a_{mnkl}=0$ for $k>m$ or $l>n$ or both. We also see by [9] that an infinite matrix $A=(a_{mnkl})$ is said to be a triangular if $a_{mnmn}\neq0$ for all $m,n\in\mathbb{N}$. Moreover, Cooke [10] showed that every triangular matrix has a unique inverse which is also a triangular matrix.

The four-dimensional generalized difference matrix $B(r,s,t,u)=\{ b_{mnkl}(r,s,t,u)\}$ and matrix domain of it on some double sequence spaces was recently defined and studied by Tuǧ and Başar [1], and Tuǧ [2]. The matrix $B(r,s,t,u)=\{ b_{mnkl}(r,s,t,u)\}$ was defined by

$$\begin{aligned} b_{mnkl}(r,s,t,u):= \textstyle\begin{cases} su& (k,l)=(m-1,n-1), \\ st& (k,l)=(m-1,n), \\ ru& (k,l)=(m,n-1), \\ rt& (k,l)=(m,n) \\ 0& \text{otherwise,} \end{cases}\displaystyle \end{aligned}$$

for $r,s,t,u\in\mathbb{R}\backslash\{0\}$ and for all $m,n,k,l\in \mathbb{N}$. Therefore, the $B(r,s,t,u)$-transform of a double sequence $x=(x_{mn})$ was defined by

1.2 $$\begin{aligned} y_{mn}&:=\bigl\{ B(r,s,t,u)x\bigr\} _{mn}=\sum _{k,l}b_{mnkl}(r,s,t,u)x_{kl} \\ &=sux_{m-1,n-1}+stx_{m-1,n}+rux_{m,n-1}+rtx_{mn} \end{aligned}$$

for all $m,n\in\mathbb{N}$. Moreover, the matrix $B^{-1}(r,s,t,u)=F(r,s,t,u)=\{f_{mnkl}(r,s,t,u)\}$, which is the inverse of the matrix $B(r,s,t,u)$, was calculated to be

$$\begin{aligned} f_{mnkl}(r,s,t,u):=\textstyle\begin{cases} \frac{(-s/r)^{m-k}(-u/t)^{n-l}}{rt}& 0\leq k\leq m, 0\leq l\leq n, \\ 0& \text{otherwise,} \end{cases}\displaystyle \end{aligned}$$

for all $m,n,k,l\in\mathbb{N}$. Furthermore, Tuǧ and Başar [1] obtained the relation between $x=(x_{mn})$ and $y=(y_{mn})$:

1.3 $$\begin{aligned} x_{mn}=\frac{1}{rt}\sum _{k,l=0}^{m,n} \biggl(\frac{-s}{r} \biggr)^{m-k} \biggl(\frac{-u}{t} \biggr)^{n-l}y_{kl} \quad\text{for all } m,n\in\mathbb{N}. \end{aligned}$$

In this paper, as natural continuation of [2] and [11], we introduce new almost null and almost convergent double sequence spaces $B(\mathcal{C}_{f})$ and $B(\mathcal{C}_{f_{0}})$ as the domain of four-dimensional generalized difference matrix $B(r,s,t,u)$ in the spaces $\mathcal{C}_{f}$ and $\mathcal{C}_{f_{0}}$, respectively. Throughout the paper, we suppose that the terms of the double sequence $x=(x_{mn})$ and $y=(y_{mn})$ are connected with equation (1.3) and the four-dimensional generalized difference matrix $B(r,s,t,u)=(b_{mnkl}(r,s,t,u))$ will be presented with $B=(b_{mnkl})$.

## The sequence space $\mathcal{C}_{f}$ of almost convergent double sequences

Lorentz [12] introduced the concept of almost convergence for a single sequence and Móricz and Rhoades [13] extended and studied this concept for a double sequence. A double sequence $x=(x_{kl})$ of complex numbers is said to be almost convergent to a generalized limit *L* if

$$\begin{aligned} p-\lim_{q,q'\to\infty}\sup_{m,n>0} \Biggl\vert \frac{1}{(q+1)(q'+1)}\sum_{k=m}^{m+q}\sum _{l=n}^{n+q'}x_{kl}-L \Biggr\vert =0. \end{aligned}$$

In this case, *L* is called the $f_{2}$-limit of the double sequence *x*. Throughout the paper, $\mathcal{C}_{f}$ denotes the space of all almost convergent double sequences, *i.e.*,

$$\begin{aligned} \mathcal{C}_{f}:={}& \Biggl\{ x=(x_{kl})\in\Omega:\exists L \in\mathbb {C} \ni \\ & p-\lim_{q,q'\to\infty}\sup_{m,n>0} \Biggl\vert \frac {1}{(q+1)(q'+1)}\sum_{k=m}^{m+q}\sum _{l=n}^{n+q'}x_{kl}-L \Biggr\vert =0, \text{ uniformly in }m,n \Biggr\} . \end{aligned}$$

It is well known that a convergent double sequence need not be almost convergent. But it is well known that every bounded convergent double sequence is also almost convergent and every almost convergent double sequence is bounded. That is, the inclusion $\mathcal{C}_{bp}\subset \mathcal{C}_{f}\subset\mathcal{M}_{u}$ holds, and each inclusion is proper. A double sequence $x=(x_{kl})$ is called almost Cauchy, which was introduced by Čunjalo [14], if for every $\epsilon>0$ there exists a positive integer *K* such that

$$\begin{aligned} \Biggl\vert \frac{1}{(q_{1}+1)(q_{1}'+1)}\sum_{k=m_{1}}^{m_{1}+q_{1}} \sum_{l=n_{1}}^{n_{1}+q_{1}'}x_{kl}- \frac{1}{(q_{2}+1)(q_{2}'+1)}\sum_{k=m_{2}}^{m_{2}+q_{2}}\sum _{l=n_{2}}^{n_{2}+q_{2}'}x_{kl} \Biggr\vert < \epsilon \end{aligned}$$

for all $q_{1}, q_{1}', q_{2}, q_{2}'>K$ and $(m_{1},n_{1}),(m_{2},n_{2})\in\mathbb {N}\times\mathbb{N}$. Mursaleen and Mohiuddine [15] proved that every double sequence is almost convergent if and only if it is almost Cauchy.

Móricz and Rhoades [13] considered that four-dimensional matrices transforming every almost convergent double sequence into a *bp*-convergent double sequence with the same limit. Almost conservative and almost regular matrices for single sequences were characterized by King [16] and almost $\mathcal{C}_{\vartheta }$-conservative and almost $\mathcal{C}_{\vartheta}$-regular four-dimensional matrices for double sequences were defined and characterized by Zeltser *et al.* [17]. Mursaleen [18] introduced the almost strongly regularity for double sequences. A four-dimensional matrix $A=(a_{mnkl})$ is called almost strongly regular if it transforms every almost convergent double sequence into an almost convergent double sequence with the same limit.

### Definition 2.1

([17])

A four-dimensional matrix $A=(a_{mnkl})$ is said to be almost $\mathcal {C}_{\vartheta}$-conservative matrix if it transforms every *ϑ*-convergent double sequence $x=(x_{kl})$ into an almost convergent double sequence space, that is, $A=(a_{mnkl})\in(\mathcal {C}_{\vartheta}:\mathcal{C}_{f})$.

### Definition 2.2

([17])

A four-dimensional matrix $A=(a_{mnkl})$ is said to be almost $\mathcal {C}_{\vartheta}$-regular if it is $\mathcal{C}_{\vartheta }$-conservative and $f_{2}-\lim Ax=\vartheta-\lim x$ for all $x\in \mathcal{C}_{\vartheta}$.

## Spaces of almost B-summable double sequences

In this present section, we define new almost convergent double sequence spaces $B(\mathcal{C}_{f})$ and $B(\mathcal{C}_{f_{0}})$ derived by the domain of four-dimensional matrix *B* in the spaces of all almost convergent and almost null double sequences $\mathcal{C}_{f}$ and $\mathcal{C}_{f_{0}}$, respectively. Then we show that $B(\mathcal {C}_{f})$ and $B(\mathcal{C}_{f_{0}})$ are Banach spaces with the norm $\Vert x \Vert _{B(\mathcal{C}_{f})}$, and we prove an inclusion relation.

Now we may define the spaces $B(\mathcal{C}_{f})$ and $B(\mathcal {C}_{f_{0}})$ by

$$\begin{aligned} B(\mathcal{C}_{f}):={}& \Biggl\{ x=(x_{kl})\in\Omega:\exists L\in \mathbb{C} \ni \\ &p-\lim_{q,q'\to\infty}\sup_{m,n>0} \Biggl\vert \frac {1}{(q+1)(q'+1)}\sum_{k=m}^{m+q}\sum _{l=n}^{n+q'}(Bx)_{kl}-L \Biggr\vert =0, \text{ uniformly in }m,n \Biggr\} , \\ B(\mathcal{C}_{f_{0}}):= {}&\Biggl\{ x=(x_{kl})\in\Omega: \\ &p-\lim_{q,q'\to\infty}\sup_{m,n>0} \Biggl\vert \frac {1}{(q+1)(q'+1)}\sum_{k=m}^{m+q}\sum _{l=n}^{n+q'}(Bx)_{kl} \Biggr\vert =0, \text{ uniformly in }m,n \Biggr\} . \end{aligned}$$

### Theorem 3.1

*The sequence spaces* $B(\mathcal{C}_{f})$ *and* $B(\mathcal{C}_{f_{0}})$ *are Banach spaces and linearly norm isomorphic to the spaces* $\mathcal {C}_{f}$ *and* $\mathcal{C}_{f_{0}}$, *respectively*, *with the norm defined by*

3.1 $$\begin{aligned} \Vert x \Vert _{B(\mathcal{C}_{f})}=\sup_{q,q',m,n\in\mathbb{N}} \Biggl\vert \frac {1}{(q+1)(q'+1)}\sum_{k=m}^{m+q} \sum_{l=n}^{n+q'}(Bx)_{kl} \Biggr\vert . \end{aligned}$$

### Proof

Since in other cases it can be similarly proved, we prove the theorem only for the space $B(\mathcal{C}_{f})$. Let us consider a Cauchy sequence $x^{(j)}=\{x_{kl}^{(j)}\}_{k,l\in\mathbb{N}}\in B(\mathcal {C}_{f})$. Then, for a given $\epsilon>0$, there exists a positive integer $M(\epsilon)\in\mathbb{N}$ such that

3.2 $$\begin{aligned} \bigl\Vert x^{(j)}-x^{(i)} \bigr\Vert _{B(\mathcal{C}_{f})}&= \sup_{q,q',m,n\in\mathbb {N}} \Biggl\vert \frac{1}{(q+1)(q'+1)}\sum _{k=m}^{m+q}\sum_{l=n}^{n+q'} \bigl[\bigl(Bx^{(j)}\bigr)_{kl}-\bigl(Bx^{(i)} \bigr)_{kl} \bigr] \Biggr\vert \\ &< \epsilon \end{aligned}$$

for all $i,j>M(\epsilon)$. Then we can read from equation (3.2) that $\{(Bx^{(j)})_{kl}\}_{j\in\mathbb{N}}$ is Cauchy in $\mathcal{C}_{f}$ for each $k,l\in\mathbb{N}$. Since $\mathcal{C}_{f}$ is complete with the norm $\Vert x \Vert _{\mathcal{C}_{f}}$ (see [19]), it is convergent. Then we may say that there exists a double sequence $x=(x_{kl})\in\mathcal{C}_{f}$ such that

$$\begin{aligned} \frac{1}{(q+1)(q'+1)}\sum_{k=m}^{m+q}\sum _{l=n}^{n+q'}\bigl(Bx^{(j)} \bigr)_{kl}\to\frac{1}{(q+1)(q'+1)}\sum_{k=m}^{m+q} \sum_{l=n}^{n+q'}(Bx)_{kl} \end{aligned}$$

as $j\to\infty$. Now, by taking the limit as $i\to\infty$ on the equality (3.2), for every $\epsilon>0$ we have for all $k,l\in\mathbb{N}$

$$\begin{aligned} \Biggl\vert \frac{1}{(q+1)(q'+1)}\sum_{k=m}^{m+q} \sum_{l=n}^{n+q'}\bigl(Bx^{(j)} \bigr)_{kl}-\frac{1}{(q+1)(q'+1)}\sum_{k=m}^{m+q} \sum_{l=n}^{n+q'}(Bx)_{kl} \Biggr\vert < \epsilon. \end{aligned}$$

Moreover, since $\{(Bx^{(j)})_{kl}\}\in\mathcal{C}_{f}$ and every almost convergent double sequence is bounded, there exists a positive real number *K* such that

$$\begin{aligned} \sup_{m,n\in\mathbb{N}} \Biggl\vert \frac{1}{(q+1)(q'+1)}\sum _{k=m}^{m+q}\sum_{l=n}^{n+q'} \bigl(Bx^{(j)}\bigr)_{kl} \Biggr\vert \leq K. \end{aligned}$$

Therefore, we are enabled to write the following inequality:

$$\begin{aligned} \Biggl\vert \frac{1}{(q+1)(q'+1)}\sum_{k=m}^{m+q} \sum_{l=n}^{n+q'}(Bx)_{kl} \Biggr\vert \leq{}& \Biggl\vert \frac{1}{(q+1)(q'+1)}\sum_{k=m}^{m+q} \sum_{l=n}^{n+q'}\bigl(Bx^{(j)} \bigr)_{kl} \\ &{}-\frac{1}{(q+1)(q'+1)}\sum_{k=m}^{m+q} \sum_{l=n}^{n+q'}(Bx)_{kl} \Biggr\vert \\ &{}+ \Biggl\vert \frac{1}{(q+1)(q'+1)}\sum_{k=m}^{m+q} \sum_{l=n}^{n+q'}\bigl(Bx^{(j)} \bigr)_{kl} \Biggr\vert \\ < {}&\epsilon+K. \end{aligned}$$

Now we can say by taking the supremum over $m,n\in\mathbb{N}$ and the *p*-limit as $q,q'\to\infty$ from the inequality acquired above that

$$\begin{aligned} \Biggl(\sum_{k=m}^{m+q}\sum _{l=n}^{n+q'}(Bx)_{kl}/\bigl[(q+1) \bigl(q'+1\bigr)\bigr] \Biggr)\in\mathcal{C}_{f}, \end{aligned}$$

that is, $x\in B(\mathcal{C}_{f})$. We see from this approach that the space $B(\mathcal{C}_{f})$ is a Banach space with the norm $\Vert \cdot \Vert _{B(\mathcal{C}_{f})}$ defined by (3.1).

Now, we should show here that $B(\mathcal{C}_{f})\cong\mathcal{C}_{f}$. To show this, we should prove the existence of a linear bijection between the spaces $B(\mathcal{C}_{f})$ and $\mathcal{C}_{f}$. Let us consider the transformation *T* from $B(\mathcal{C}_{f})$ to $\mathcal {C}_{f}$ by $x\mapsto Tx=y=Bx$, with the notation of (1.2). The linearity and injectivity of *T* is clear. Let us take any $y=(y_{kl})\in\mathcal{C}_{f}$ and consider the sequence $x=(x_{kl})$ with respect to the sequence *y* by equation (1.3) for all $k,l\in\mathbb{N}$. Then we have the following equality:

$$\begin{aligned} (Bx)_{kl}={}&sux_{k-1,l-1}+stx_{k-1,l}+rux_{k,l-1}+rtx_{kl} \\ ={}&su\sum_{i,j=0}^{k-1,l-1} \biggl( \frac{-s}{r} \biggr)^{k-i-1} \biggl(\frac{-u}{t} \biggr)^{l-j-1}\frac{y_{ij}}{rt} \\ &{}+st\sum_{i,j=0}^{k-1,l} \biggl( \frac{-s}{r} \biggr)^{k-i-1} \biggl(\frac{-u}{t} \biggr)^{l-j}\frac{y_{ij}}{rt} \\ &{}+ ru\sum_{i,j=0}^{k,l-1} \biggl( \frac{-s}{r} \biggr)^{k-i} \biggl(\frac{-u}{t} \biggr)^{l-j-1}\frac{y_{ij}}{rt} \\ &{}+ rt\sum_{i,j=0}^{k,l} \biggl( \frac{-s}{r} \biggr)^{k-i} \biggl(\frac {-u}{t} \biggr)^{l-j}\frac{y_{ij}}{rt} \\ ={}&y_{kl} \end{aligned}$$

for all $k,l\in\mathbb{N}$. Thus, we arrive at the consequence that

$$\begin{aligned} p-\lim_{q,q'\to\infty}\sum_{k=m}^{m+q} \sum_{l=n}^{n+q'}(Bx)_{kl}/ \bigl[(q+1) \bigl(q'+1\bigr)\bigr]=p-\lim_{q,q'\to\infty}\sum _{k=m}^{m+q}\sum_{l=n}^{n+q'}y_{kl}/ \bigl[(q+1) \bigl(q'+1\bigr)\bigr]. \end{aligned}$$

This shows that $x=(x_{kl})\in B(\mathcal{C}_{f})$. Then we may say that *T* is surjective. Moreover, one can obtain the following equality:

$$\begin{aligned} \Vert x \Vert _{B(\mathcal{C}_{f})}&=\sup_{q,q',m,n\in\mathbb{N}} \Biggl\vert \frac {1}{(q+1)(q'+1)}\sum_{k=m}^{m+q}\sum _{l=n}^{n+q'}(Bx)_{kl} \Biggr\vert \\ &=\sup_{q,q',m,n\in\mathbb{N}} \Biggl\vert \frac{1}{(q+1)(q'+1)}\sum _{k=m}^{m+q}\sum_{l=n}^{n+q'}y_{kl} \Biggr\vert = \Vert y \Vert _{\mathcal {C}_{f}}< \infty. \end{aligned}$$

That is, *T* is norm preserving. Hence, *T* is linear bijection and $B(\mathcal{C}_{f})$ and $\mathcal{C}_{f}$ are linearly norm isomorphic. This is what we proposed. □

### Theorem 3.2

*Let* $s=-r, t=-u$. *The inclusions* $\mathcal{C}_{f}\subset B(\mathcal {C}_{f})$ *and* $\mathcal{C}_{f_{0}}\subset B(\mathcal{C}_{f_{0}})$ *strictly hold*.

### Proof

Firstly, we should prove that the inclusions $\mathcal{C}_{f}\subset B(\mathcal{C}_{f})$ and $\mathcal{C}_{f_{0}}\subset B(\mathcal {C}_{f_{0}})$ hold. Since $s=-r, t=-u$, the four-dimensional matrix $B=(b_{mnkl})$ satisfy the conditions of Lemma 4.8. Then we can say that, for all $x\in\mathcal{C}_{f}\ (\text{or }\mathcal {C}_{f_{0}}), Bx\in\mathcal{C}_{f}\ (\text{or }\mathcal{C}_{f_{0}})$ whenever $x\in B(\mathcal{C}_{f})\ (\text{or }B(\mathcal{C}_{f_{0}}))$, which means that the inclusions $\mathcal{C}_{f}\subset B(\mathcal {C}_{f})$ and $\mathcal{C}_{f_{0}}\subset B(\mathcal{C}_{f_{0}})$ hold.

In order to show that the inclusions are strict, we should show that the sets $B(\mathcal{C}_{f})\setminus\mathcal{C}_{f}$ and $B(\mathcal {C}_{f_{0}})\setminus\mathcal{C}_{f_{0}}$ are not empty, that is, there exists a double sequence $x=(x_{mn})$ which belongs to $B(\mathcal {C}_{f})$ but not in $\mathcal{C}_{f}$. Let consider a double sequence $x=(x_{mn})$ by $x_{mn}=\frac{mn}{rt}$ for all $m,n\in\mathbb{N}$. Since it is not bounded, it is obvious that $x\notin\mathcal{C}_{f}$. But if $s=-r, t=-u$, then we obtain the *B*-transform of *x* as

$$\begin{aligned} (Bx)_{mn}=\bigl\{ B(r,-r,t,-t)x\bigr\} _{mn}={}& rtx_{m-1,n-1}-rtx_{m-1,n}-rtx_{m,n-1}+rtx_{mn} \\ = {}&rt\frac{(m-1)(n-1)}{rt}-rt\frac{(m-1)n}{rt} \\ &{}-rt\frac{m(n-1)}{rt}+rt\frac{mn}{rt} \\ ={}& 1. \end{aligned}$$

Therefore, we have the following equality with the above result:

$$\begin{aligned} \frac{1}{(q+1)(q'+1)} \Biggl\vert \sum_{k=m}^{m+q} \sum_{l=n}^{n+q'}(Bx)_{kl} \Biggr\vert =1. \end{aligned}$$

After taking the supremum over $m,n\in\mathbb{N}$ in the above equality and applying the *p*-limit as $q,q'\to\infty$ we see that $Bx\in\mathcal{C}_{f}$. It can easily be shown that the sequence $x_{mn}=\frac{m}{rt}$ for all $m,n\in\mathbb{N}$ is in $B(\mathcal{C}_{f_{0}})\setminus\mathcal {C}_{f_{0}}$ by the same reasoning as above. So we omit the details. □

## The *α*-, $\beta(\vartheta)$- and *γ*-duals of the sequence space $B(\mathcal{C}_{f})$

In this section, firstly, we calculate the *α*-dual of the space $B(\mathcal{C}_{f})$. Then we give some known definitions and lemmas which will be used in the proof of $\beta(bp)$-dual of the space $B(\mathcal{C}_{f})$ and in the fourth section of this paper. Moreover, we characterize a new four-dimensional matrix class $(\mathcal {C}_{f}:\mathcal{M}_{u})$ in order to calculate the *γ*-dual of the space $B(\mathcal{C}_{f})$.

### Theorem 4.1

*Let* $\vert s/r \vert , \vert u/t \vert <1$. *The* *α*-*dual of the space* $B(\mathcal{C}_{f})$ *is the space* $\mathcal{L}_{u}$.

### Proof

We shall prove that the equality $\{B(\mathcal{C}_{f}) \} ^{\alpha}=\mathcal{L}_{u}$ and that the inclusions $\mathcal {L}_{u}\subset \{B(\mathcal{C}_{f}) \}^{\alpha}$ and $\{ B(\mathcal{C}_{f}) \}^{\alpha}\subset\mathcal{L}_{u}$ hold. For the first inclusion, let us take a sequence $a=(a_{kl})\in\mathcal {L}_{u}$ and $x=(x_{kl})\in B(\mathcal{C}_{f})$. Then there exists a double sequence $y=(y_{kl})\in\mathcal{C}_{f}$ with equation (1.2) such that

$$\begin{aligned} p-\lim_{q,q'\to\infty}\sup_{m,n>0} \Biggl\vert \frac{1}{(q+1)(q'+1)}\sum_{k=m}^{m+q}\sum _{l=n}^{n+q'}y_{kl} \Biggr\vert \quad \text{exists.} \end{aligned}$$

Moreover, the inclusion $\mathcal{C}_{f}\subset\mathcal{M}_{u}$ holds, and there exists a positive real number *K* such that $\sup_{k,l} \vert y_{kl} \vert \leq K$. Since $\vert s/r \vert , \vert u/t \vert <1$, we have the following inequality:

$$\begin{aligned} \sum_{k,l} \vert a_{kl}x_{kl} \vert &=\sum_{k,l} \vert a_{kl} \vert \Biggl\vert \sum_{i,j=0}^{k,l} \biggl( \frac{-s}{r} \biggr)^{k-i} \biggl(\frac {-u}{t} \biggr)^{l-j}\frac{y_{ij}}{rt} \Biggr\vert \\ &\leq\frac{1}{ \vert rt \vert }\sum_{k,l} \vert a_{kl} \vert \sum_{i,j=0}^{k,l} \biggl\vert \biggl(\frac{-s}{r} \biggr)^{k-i} \biggl( \frac {-u}{t} \biggr)^{l-j} \biggr\vert \vert y_{ij} \vert \\ &\leq\frac{K}{ \vert rt \vert }\sum_{k,l} \vert a_{kl} \vert \sum_{i,j=0}^{k,l} \biggl\vert \frac{-s}{r} \biggr\vert ^{k-i} \biggl\vert \frac {-u}{t} \biggr\vert ^{l-j} \\ &=\frac{K}{ \vert rt \vert }\sum_{k,l} \vert a_{kl} \vert \biggl(\frac{1- \vert \frac{s}{r} \vert ^{k+1}}{1- \vert \frac{s}{r} \vert } \biggr) \biggl( \frac{1- \vert \frac{u}{t} \vert ^{l+1}}{1- \vert \frac {u}{t} \vert } \biggr) \\ &=\frac{K}{ \vert rt \vert } \biggl(\frac{1}{1- \vert \frac{s}{r} \vert } \biggr) \biggl( \frac{1}{1- \vert \frac{u}{t} \vert } \biggr)\sum_{k,l} \vert a_{kl} \vert \biggl(1- \biggl\vert \frac{s}{r} \biggr\vert ^{k+1} \biggr) \biggl(1- \biggl\vert \frac{u}{t} \biggr\vert ^{l+1} \biggr) \\ &\leq \frac{K}{ \vert rt \vert } \biggl(\frac{1}{1- \vert \frac{s}{r} \vert } \biggr) \biggl( \frac{1}{1- \vert \frac{u}{t} \vert } \biggr)\sum_{k,l} \vert a_{kl} \vert \\ &< \infty, \end{aligned}$$

which says that $a=(a_{kl})\in \{B(\mathcal{C}_{f}) \} ^{\alpha}$. Hence, the inclusion $\mathcal{L}_{u}\subset \{ B(\mathcal{C}_{f}) \}^{\alpha}$ holds.

Conversely, suppose that $(a_{kl})\in \{B(\mathcal{C}_{f}) \} ^{\alpha}\setminus\mathcal{L}_{u}$. Then we have $\sum_{k,l} \vert a_{kl}x_{kl} \vert <\infty$ for all $x=(x_{kl})\in B(\mathcal {C}_{f})$. When we define a double sequence $x=(x_{kl})$ in the special case of $x=(x_{kl})=\{(-1)^{k+l}/(rt)\}$ for $r=\alpha s$, $t=\alpha u$ and $\alpha\in\mathbb{R}-[-1,1]$, it is trivial to see that $x=(x_{kl})=\{(-1)^{k+l}/(rt)\}\in B(\mathcal{C}_{f})$ but

$$\begin{aligned} \sum_{k,l} \vert a_{kl}x_{kl} \vert =\frac{1}{ \vert rt \vert }\sum_{k,l} \vert a_{kl} \vert =\infty. \end{aligned}$$

This means that $(a_{kl})\notin \{B(\mathcal{C}_{f}) \} ^{\alpha}$, which is a contradiction. Therefore, $(a_{kl})$ must belong to the space $\mathcal{L}_{u}$. So, the inclusion $\{ B(\mathcal{C}_{f}) \}^{\alpha}\subset\mathcal{L}_{u}$ holds. This completes the proof. □

Now we have the following significant lemmas, which will be used in this present section and the fifth section of this work.

### Lemma 4.2

([17])

*The following statements hold*:

(a) *A four*-*dimensional matrix* $A=(a_{mnkl})$ *is almost* $\mathcal{C}_{bp}$-*conservative*, *i*.*e*., $A\in(\mathcal {C}_{bp}:\mathcal{C}_{f})$, *iff the following conditions hold*:

4.1 $$\begin{aligned} &\sup_{m,n\in\mathbb{N}}\sum_{k,l} \vert a_{mnkl} \vert < \infty , \end{aligned}$$

4.2 $$\begin{aligned} &\exists a_{ij}\in\mathbb{C} \ni bp-\lim _{q,q'\to \infty}a\bigl(i,j,q,q',m,n\bigr)=a_{ij}, \\ &\quad \textit{uniformly in } m,n\in\mathbb{N}\textit{ for each }i,j\in \mathbb{N,} \end{aligned}$$

4.3 $$\begin{aligned} &\exists u\in\mathbb{C} \ni bp-\lim_{q,q'\to\infty }\sum _{i,j}a\bigl(i,j,q,q',m,n\bigr)=u, \\ &\quad\textit{uniformly in } m,n\in\mathbb{N,} \end{aligned}$$

4.4 $$\begin{aligned} &\exists a_{ij}\in\mathbb{C} \ni bp-\lim _{q,q'\to \infty}\sum_{i} \bigl\vert a \bigl(i,j,q,q',m,n\bigr)-a_{ij} \bigr\vert =0, \\ &\quad\textit{uniformly in } m,n\in\mathbb{N}\textit{ for each }j\in \mathbb{N,} \end{aligned}$$

4.5 $$\begin{aligned} &\exists a_{ij}\in\mathbb{C} \ni bp-\lim _{q,q'\to \infty}\sum_{j} \bigl\vert a \bigl(i,j,q,q',m,n\bigr)-a_{ij} \bigr\vert =0, \\ &\quad\textit{uniformly in } m,n\in\mathbb{N}\textit{ for each }i\in \mathbb{N,} \end{aligned}$$

*where* $a(i,j,q,q',m,n)=\sum_{k=m}^{m+q}\sum_{l=n}^{n+q'}a_{klij}/[(q+1)(q'+1)]$. *In this case*, $a=(a_{ij})\in \mathcal{L}_{u}$ *and*

$$\begin{aligned} f_{2}-\lim Ax=\sum_{i,j}a_{ij}x_{ij}+ \biggl(u-\sum_{i,j}a_{ij} \biggr)bp-\lim _{i,j\to\infty}x_{ij}, \end{aligned}$$

*that is*,

$$\begin{aligned} &bp-\lim_{q,q'\to\infty}\sum_{i,j}a \bigl(i,j,q,q',m,n\bigr)x_{ij}=\sum _{i,j}a_{ij}x_{ij}+ \biggl(u-\sum _{i,j}a_{ij} \biggr)bp-\lim_{i,j\to \infty}x_{ij}, \\ &\textit{ uniformly in }m,n\in\mathbb{N}. \end{aligned}$$

(b) *A four*-*dimensional matrix* $A=(a_{mnkl})$ *is almost* $\mathcal{C}_{bp}$-*regular*, *i*.*e*., $A\in(\mathcal{C}_{bp}:\mathcal {C}_{f})_{\mathrm{reg}}$, *iff the conditions* (4.1)-(4.5) *hold with* $a_{ij}=0$ *for all* $i,j\in\mathbb{N}$ *and* $u=1$

### Lemma 4.3

([17])

*The following statements hold*:

- *A four*-*dimensional matrix* $A=(a_{mnkl})$ *is almost* $\mathcal{C}_{r}$-*conservative*, *i*.*e*., $A\in(\mathcal{C}_{r}:\mathcal {C}_{f})$ *iff* (4.1)-(4.3) *and the following conditions hold*:
  4.6 $$\begin{aligned} &\exists j_{0}\in\mathbb{N} \ni bp-\lim _{q,q'\to \infty}\sum_{i}a \bigl(i,j_{0},q,q',m,n\bigr)=u_{j_{0}}, \\ &\quad\textit{uniformly in } m,n\in\mathbb{N}, \end{aligned}$$
  4.7 $$\begin{aligned} &\exists i_{0}\in\mathbb{N} \ni bp-\lim _{q,q'\to \infty}\sum_{j}a \bigl(i_{0},j,q,q',m,n\bigr)=v_{i_{0}}, \\ &\quad\textit{uniformly in } m,n\in\mathbb{N}, \end{aligned}$$
  *where* $a(i,j,q,q',m,n)$ *is defined as in Lemma *4.2. *In this case*, $a=(a_{ij})\in\mathcal{L}_{u}$; $(u_{j}),(v_{i})\in\ell_{1}$ *and*
  $$\begin{aligned} f_{2}-\lim Ax ={}&\sum_{ij}a_{ij}x_{ij}+ \sum_{i} \biggl(v_{i}-\sum _{j}a_{ij} \biggr)x_{i}+\sum _{j} \biggl(u_{j}-\sum _{i}a_{ij} \biggr)x_{j} \\ &{}+ \biggl(u+\sum_{i,j}a_{ij}-\sum _{i}v_{i}-\sum _{j}u_{j} \biggr) r-\lim_{i,j\to\infty}x_{ij}. \end{aligned}$$
- *A four*-*dimensional matrix* $A=(a_{mnkl})$ *is almost* $\mathcal{C}_{r}$-*regular*, *i*.*e*., $A\in(\mathcal{C}_{r}:\mathcal {C}_{f})_{\mathrm{reg}}$, *iff the conditions* (4.1)-(4.7) *hold with* $a_{ij}=u_{j}=v_{i}=0$ *for all* $i,j\in\mathbb{N}$ *and* $u=1$.

### Lemma 4.4

([17])

*The following statements hold*:

- *A four*-*dimensional matrix* $A=(a_{mnkl})$ *is almost* $\mathcal{C}_{p}$-*conservative*, *i*.*e*., $A\in(\mathcal{C}_{p}:\mathcal {C}_{f})$, *iff* (4.1)-(4.3) *hold*. *In this case* $a=(a_{ij})\in\mathcal{L}_{u}, (a_{ij_{0}})_{i\in\mathbb {N}}, (a_{i_{0}j})_{j\in\mathbb{N}}\in\varphi$ *where* *φ* *denotes the space of all finitely non*-*zero sequences and*
  $$\begin{aligned} f_{2}-\lim Ax=\sum_{i,j}a_{ij}x_{ij}+ \biggl(u-\sum_{i,j}a_{ij} \biggr)p-\lim _{i,j\to\infty}x_{ij}. \end{aligned}$$
- *A four*-*dimensional matrix* $A=(a_{mnkl})$ *is almost* $\mathcal{C}_{p}$-*regular*, *i*.*e*., $A\in(\mathcal{C}_{r}:\mathcal {C}_{f})_{\mathrm{reg}}$, *iff the conditions* (4.1)-(4.3) *hold with* $a_{ij}=0$ *for all* $i,j\in\mathbb{N}$ *and* $u=1$.

### Lemma 4.5

([13])

*The following statements hold*:

- *A four*-*dimensional matrix* $A=(a_{mnkl})\in(\mathcal {C}_{f}:\mathcal{C}_{bp})$ *iff the condition* (4.1) *and the following conditions hold*:
  4.8 $$\begin{aligned} &\exists a_{kl}\in\mathbb{C}\ni, bp-\lim _{m,n\to \infty}a_{mnkl}=a_{kl}\quad\textit{for all } k,l \in\mathbb{N}, \end{aligned}$$
  4.9 $$\begin{aligned} &\exists u\in\mathbb{C}\ni, bp-\lim_{m,n\to\infty }\sum _{k,l}a_{mnkl}=u, \end{aligned}$$
  4.10 $$\begin{aligned} &\exists k_{0}\in\mathbb{N}\ni, bp-\lim _{m,n\to \infty}\sum_{l} \vert a_{mn,k_{0},l}-a_{k_{0},l} \vert =0\quad\textit{for all } l\in \mathbb{N}, \end{aligned}$$
  4.11 $$\begin{aligned} &\exists l_{0}\in\mathbb{N}\ni, bp-\lim _{m,n\to \infty}\sum_{k} \vert a_{mnk,l_{0}}-a_{k,l_{0}} \vert =0\quad\textit{for all } k\in \mathbb{N}, \end{aligned}$$
  4.12 $$\begin{aligned} &bp-\lim_{m,n\to\infty}\sum _{k}\sum_{l} \vert \Delta _{01}a_{mnkl} \vert =0, \end{aligned}$$
  4.13 $$\begin{aligned} &bp-\lim_{m,n\to\infty}\sum _{k}\sum_{l} \vert \Delta _{10}a_{mnkl} \vert =0. \end{aligned}$$
- *A four*-*dimensional matrix* $A=(a_{mnkl})$ *is strongly regular*, *i*.*e*., $A\in(\mathcal{C}_{f}:\mathcal{C}_{bp})_{\mathrm{reg}}$, *iff the conditions* (4.1) *and* (4.8)-(4.13) *hold with* $a_{kl}=0$ *for all* $k,l\in\mathbb{N}$ *and* $u=1$, *where* $\Delta_{10}a_{mnkl}=a_{mnkl}-a_{m,n,k+1,l}$ *and* $\Delta _{01}a_{mnkl}=a_{mnkl}-a_{m,n,k,l+1}$, $(m,n,k,l=0,1,2,\ldots)$.

### Lemma 4.6

([20])

*The four*-*dimensional matrix* $A=(a_{mnkl})\in(\mathcal{M}_{u}:\mathcal {C}_{f})$ *iff the condition* (4.1) *and the following conditions hold*:

4.14 $$\begin{aligned} &\exists\beta_{kl}\in\mathbb{C}\ni f_{2}- \lim_{m,n\to\infty}a_{mnkl}=\beta_{kl} \quad\textit{for all } k,l\in \mathbb{N}, \end{aligned}$$

4.15 $$\begin{aligned} &\textit{for every }m,n,j\in\mathbb{N}, \exists K\in \mathbb{N} \ni\frac{1}{(q+1)(q'+1)}\sum_{k=m}^{m+q}\sum _{l=n}^{n+q'}a_{klij}=0, \\ &\quad\textit{for all }q,q',i>K, \end{aligned}$$

4.16 $$\begin{aligned} &\textit{for every }m,n,i\in\mathbb{N}, \exists L\in \mathbb{N} \ni\frac{1}{(q+1)(q'+1)}\sum_{k=m}^{m+q}\sum _{l=n}^{n+q'}a_{klij}=0, \\ &\quad\textit{for all }q,q',j>L. \end{aligned}$$

### Lemma 4.7

([21])

*A four*-*dimensional matrix* $A=(a_{mnkl})$ *is almost regular*, *i*.*e*., $A\in (\mathcal{C}_{bp}:\mathcal{C}_{f})_{\mathrm{reg}}$, *iff the condition* (4.1) *and the following conditions hold*:

4.17 $$\begin{aligned} &\lim_{q,q'\to\infty}a\bigl(i,j,q,q',m,n \bigr)=0, \\ &\quad\textit{uniformly in } m,n\in\mathbb{N}\textit{ for each }i,j\in \mathbb{N}, \end{aligned}$$

4.18 $$\begin{aligned} &\lim_{q,q'\to\infty}\sum_{i,j}a \bigl(i,j,q,q',m,n\bigr)=1, \\ &\quad\textit{uniformly in } m,n\in\mathbb{N}, \end{aligned}$$

4.19 $$\begin{aligned} &\lim_{q,q'\to\infty}\sum_{i} \bigl\vert a\bigl(i,j,q,q',m,n\bigr) \bigr\vert =0, \\ &\quad\textit{uniformly in } m,n\in\mathbb{N}\textit{ for each }j\in \mathbb{N}, \end{aligned}$$

4.20 $$\begin{aligned} &\lim_{q,q'\to\infty}\sum_{j} \bigl\vert a\bigl(i,j,q,q',m,n\bigr) \bigr\vert =0, \\ &\quad\textit{uniformly in } m,n\in\mathbb{N}\textit{ for each }i\in \mathbb{N}, \end{aligned}$$

where $a(i,j,q,q',m,n)$ is defined as in Lemma 4.2.

### Lemma 4.8

([18])

*A four*-*dimensional matrix* $A=(a_{mnkl})$ *is almost strongly regular*, *i*.*e*., $A\in(\mathcal{C}_{f}:\mathcal{C}_{f})_{\mathrm{reg}}$, *iff* *A* *is almost regular and the following two conditions hold*:

4.21 $$\begin{aligned} &\lim_{q,q'\to\infty}\sum_{i} \sum_{j} \bigl\vert \Delta _{10}a \bigl(i,j,q,q',m,n\bigr) \bigr\vert =0\quad\textit{uniformly in } m,n \in\mathbb {N}, \end{aligned}$$

4.22 $$\begin{aligned} &\lim_{q,q'\to\infty}\sum_{j} \sum_{i} \bigl\vert \Delta _{01}a \bigl(i,j,q,q',m,n\bigr) \bigr\vert =0\quad\textit{uniformly in } m,n \in\mathbb{N}, \end{aligned}$$

*where*

$$\begin{aligned} &\Delta_{10}a\bigl(i,j,q,q',m,n\bigr)=a \bigl(i,j,q,q',m,n\bigr)-a\bigl(i+1,j,q,q',m,n\bigr), \\ &\Delta_{01}a\bigl(i,j,q,q',m,n\bigr)=a \bigl(i,j,q,q',m,n\bigr)-a\bigl(i,j+1,q,q',m,n\bigr). \end{aligned}$$

Now let us define the sets $d_{k}$ with $k\in\{1,2,\ldots, 7\}$, for the compressing of the following theorems and their proofs, as follows:

$$\begin{aligned} d_{1}= {}&\Biggl\{ a=(a_{kl})\in\Omega:\sup _{m,n\in\mathbb{N}}\sum_{k,l} \Biggl\vert \sum _{j,i=k,l}^{m,n} \biggl(\frac{-s}{r} \biggr)^{j-k} \biggl(\frac{-u}{t} \biggr)^{i-l} \frac{a_{ji}}{rt} \Biggr\vert < \infty \Biggr\} , \\ d_{2}={}& \Biggl\{ a=(a_{kl})\in\Omega: \\ &\exists\beta_{kl}\in\mathbb{C}\ni, \vartheta-\lim _{m,n\to \infty}\sum_{j,i=k,l}^{m,n} \biggl(\frac{-s}{r} \biggr)^{j-k} \biggl(\frac{-u}{t} \biggr)^{i-l}a_{ji}=\beta_{kl} \Biggr\} , \\ d_{3}={}& \Biggl\{ a=(a_{kl})\in\Omega: \\ &\exists u\in\mathbb{C}\ni, \vartheta-\lim_{m,n\to\infty}\sum _{k,l}\sum_{j,i=k,l}^{m,n} \biggl(\frac{-s}{r} \biggr)^{j-k} \biggl(\frac{-u}{t} \biggr)^{i-l}\frac{a_{ji}}{rt}=u \Biggr\} , \\ d_{4}={}& \Biggl\{ a=(a_{kl})\in\Omega:\exists l_{0}\in\mathbb{N}\ni, \\ &\vartheta-\lim_{m,n\to\infty}\sum_{k} \Biggl\vert \sum_{j,i=k,l_{0}}^{m,n} \biggl( \frac{-s}{r} \biggr)^{j-k} \biggl(\frac {-u}{t} \biggr)^{i-l_{0}}a_{ji}-\beta_{k,l_{0}} \Biggr\vert =0 \text{ for all }k\in\mathbb{N} \Biggr\} , \\ d_{5}={}& \Biggl\{ a=(a_{kl})\in\Omega:\exists k_{0}\in\mathbb{N}\ni, \\ &\vartheta-\lim_{m,n\to\infty}\sum_{l} \Biggl\vert \sum_{j,i=k_{0},l}^{m,n} \biggl( \frac{-s}{r} \biggr)^{j-k_{0}} \biggl(\frac {-u}{t} \biggr)^{i-l}a_{ji}-\beta_{k_{0},l} \Biggr\vert =0 \text{ for all }l\in\mathbb{N} \Biggr\} , \\ d_{6}={}& \Biggl\{ a=(a_{kl})\in\Omega: \\ &\vartheta-\lim_{m,n\to\infty}\sum_{k} \sum_{l} \Biggl\vert \Delta _{01} \Biggl\{ \sum_{j,i=k,l}^{m,n} \biggl( \frac{-s}{r} \biggr)^{j-k} \biggl(\frac{-u}{t} \biggr)^{i-l}\frac{a_{ji}}{rt} \Biggr\} \Biggr\vert =0 \Biggr\} , \\ d_{7}= {}&\Biggl\{ a=(a_{kl})\in\Omega: \\ &\vartheta-\lim_{m,n\to\infty}\sum_{k} \sum_{l} \Biggl\vert \Delta _{10} \Biggl\{ \sum_{j,i=k,l}^{m,n} \biggl( \frac{-s}{r} \biggr)^{j-k} \biggl(\frac{-u}{t} \biggr)^{i-l}\frac{a_{ji}}{rt} \Biggr\} \Biggr\vert =0 \Biggr\} . \end{aligned}$$

### Theorem 4.9

*The* $\beta(bp)$-*dual of the space* $B(\mathcal{C}_{f})$ *is the set* $\bigcap_{i=1}^{7}d_{i}$

### Proof

Suppose that $a=(a_{mn})\in\Omega$ and $x=(x_{mn})\in B(\mathcal {C}_{f})$. Then we have $y=Bx\in\mathcal{C}_{f}$. Therefore, we have the equality $(3.16)$, which is in [2, Theorem 3.11, p.14] with the $m,n$th partial sum of $\sum_{k,l}a_{kl}x_{kl}$ with $\sum_{k,l=0}^{m,n}a_{kl}x_{kl}=(Dy)_{mn}$. By taking the limit as $m,n\to \infty$ from this equality, we have the four-dimensional matrix $D=(d_{mnkl})$, which was also defined by Tuǧ [2, p. 14] as

4.23 $$\begin{aligned} d_{mnkl}=\textstyle\begin{cases} \sum_{j,i=k,l}^{m,n} (\frac{-s}{r} )^{j-k} (\frac {-u}{t} )^{i-l}\frac{a_{ji}}{rt}& 0\leq k\leq m, 0\leq l\leq n; \\ 0 & \text{otherwise,} \end{cases}\displaystyle \end{aligned}$$

for all $k,l,m,n\in\mathbb{N}$. Then one can obtain from the above consequences $ax\in\mathcal{CS}_{bp}$ whenever $x=(x_{mn})\in B(\mathcal{C}_{f})$ iff $Dy\in\mathcal{C}_{bp}$ whenever $y=(y_{mn})\in\mathcal{C}_{f}$. This says that $a=(a_{mn})\in \{ B(\mathcal{C}_{f}) \}^{\beta(\vartheta)} $ iff $D\in(\mathcal {C}_{f}: \mathcal{C}_{bp})$. Thus, we can say that the conditions of Lemma 4.5(a) hold with $d_{mnkl}$ instead of $a_{mnkl}$, i.e.,

$$\begin{aligned} &\sup_{m,n\in\mathbb{N}}\sum_{k,l} \Biggl\vert \sum_{j,i=k,l}^{m,n} \biggl( \frac{-s}{r} \biggr)^{j-k} \biggl(\frac {-u}{t} \biggr)^{i-l}\frac{a_{ji}}{rt} \Biggr\vert < \infty, \\ &\exists\beta_{kl}\in\mathbb{C}\ni, bp-\lim_{m,n\to\infty} \sum_{j,i=k,l}^{m,n} \biggl(\frac{-s}{r} \biggr)^{j-k} \biggl(\frac {-u}{t} \biggr)^{i-l}a_{ji}= \beta_{kl}, \\ &\exists u\in\mathbb{C}\ni, bp-\lim_{m,n\to\infty}\sum _{k,l}\sum_{j,i=k,l}^{m,n} \biggl(\frac{-s}{r} \biggr)^{j-k} \biggl(\frac{-u}{t} \biggr)^{i-l}\frac{a_{ji}}{rt}=u, \\ &\exists l_{0}\in\mathbb{N}\ni, bp-\lim_{m,n\to\infty}\sum _{k} \Biggl\vert \sum _{j,i=k,l_{0}}^{m,n} \biggl(\frac{-s}{r} \biggr)^{j-k} \biggl(\frac{-u}{t} \biggr)^{i-l_{0}}a_{ji}- \beta_{k,l_{0}} \Biggr\vert =0, \\ &\quad\text{for all }k\in\mathbb{N}, \\ &\exists k_{0}\in\mathbb{N}\ni, bp-\lim_{m,n\to\infty}\sum _{l} \Biggl\vert \sum _{j,i=k_{0},l}^{m,n} \biggl(\frac{-s}{r} \biggr)^{j-k_{0}} \biggl(\frac{-u}{t} \biggr)^{i-l}a_{ji}- \beta_{k_{0},l} \Biggr\vert =0, \\ &\quad\text{for all }l\in\mathbb{N}, \\ &bp-\lim_{m,n\to\infty}\sum_{k}\sum _{l} \Biggl\vert \Delta_{01} \Biggl\{ \sum_{j,i=k,l}^{m,n} \biggl(\frac{-s}{r} \biggr)^{j-k} \biggl(\frac {-u}{t} \biggr)^{i-l} \frac{a_{ji}}{rt} \Biggr\} \Biggr\vert =0, \\ &bp-\lim_{m,n\to\infty}\sum_{k}\sum _{l} \Biggl\vert \Delta_{10} \Biggl\{ \sum_{j,i=k,l}^{m,n} \biggl(\frac{-s}{r} \biggr)^{j-k} \biggl(\frac {-u}{t} \biggr)^{i-l} \frac{a_{ji}}{rt} \Biggr\} \Biggr\vert =0, \end{aligned}$$

which is the set $\bigcap_{i=1}^{7}d_{i}$. This is the result we desired. □

Now we characterize a new four-dimensional matrix class $(\mathcal {C}_{f}:\mathcal{M}_{u})$, which will be used in the proof of *γ*-dual of $\mathcal{C}_{f}$ and in some corollaries of the fifth section of this work.

### Theorem 4.10

*A four*-*dimensional matrix* $A=(a_{mnkl})\in(\mathcal{C}_{f}:\mathcal {M}_{u})$ *if and only if* $A_{mn}\in\mathcal{C}_{f}^{\beta(\vartheta)}$ *and condition* (4.1) *hold*.

### Proof

Suppose that $A=(a_{mnkl})\in(\mathcal{C}_{f}:\mathcal{M}_{u})$. Then *Ax* exists and is in $\mathcal{M}_{u}$ for all $x\in\mathcal{C}_{f}$. Then $A_{mn}\in\mathcal{C}_{f}^{\beta(\vartheta)}$ for all $m,n\in \mathbb{N}$. Moreover, it is well known from [15] that the inclusion $\mathcal{C}_{f}\subset\mathcal{M}_{u}$ holds. So, we can say that the inclusion $(\mathcal{C}_{f}:\mathcal{M}_{u})\subset(\mathcal {M}_{u}:\mathcal{M}_{u})$ holds and it gives us the result that the condition (4.1) is necessary.

Conversely, suppose that the condition (4.1) holds and $A_{mn}\in\mathcal{C}_{f}^{\beta(\vartheta)}$. Let us take any sequence $x=(x_{kl})\in\mathcal{C}_{f}\subset\mathcal{M}_{u}$, so there exists an $M\in\mathbb{R}^{+}$ such that $\sup_{k,l\in\mathbb {N}} \vert x_{kl} \vert < M$. Since $A_{mn}\in\mathcal{C}_{f}^{\beta(\vartheta)}$ for each $m,n\in\mathbb{N}$, then *Ax* exists. Since the inequality

$$\begin{aligned} \biggl\vert \sum_{k,l}a_{mnkl}x_{kl} \biggr\vert \leq\sum_{k,l} \vert a_{mnkl}x_{kl} \vert \end{aligned}$$

holds for each fixed $m,n\in\mathbb{N}$, one can obtain by taking the supremum over $m,n\in\mathbb{N}$

$$\begin{aligned} \sup_{m,n\in\mathbb{N}} \biggl\vert \sum_{k,l}a_{mnkl}x_{kl} \biggr\vert &\leq \sup_{m,n\in\mathbb{N}}\sum _{k,l} \vert a_{mnkl} \vert \vert x_{kl} \vert \\ &\leq M\sup_{m,n\in\mathbb{N}}\sum_{k,l} \vert a_{mnkl} \vert < \infty. \end{aligned}$$

This shows the fact that $Ax\in\mathcal{M}_{u}$, which completes the proof. □

### Theorem 4.11

*The* *γ*-*dual of the space* $B(\mathcal{C}_{f})$ *is the set* $d_{1}\cap CS_{\vartheta}$.

### Proof

Let us suppose that $a=(a_{mn})\in\Omega$ and $x=(x_{mn})\in B(\mathcal{C}_{f})$. Then we have $y=Bx\in\mathcal{C}_{f}$. Therefore, by following a similar way to that used in the proof of Theorem 4.9, we may say that $ax\in\mathcal{BS}$ whenever $x=(x_{mn})\in B(\mathcal{C}_{f})$ if and only if $Dy\in\mathcal{M}_{u}$ whenever $y=(y_{mn})\in\mathcal{C}_{f}$, where the matrix $D=(d_{mnkl})$ was defined by (4.23). This means that $a=(a_{mn})\in \{B(\mathcal{C}_{f}) \}^{\gamma}$ if and only if $D\in(\mathcal{C}_{f}:\mathcal{M}_{u})$. Thus, one can be seen that the conditions of Theorem 4.10 hold for the matrix $D=(d_{mnkl})$. That is, $D_{mn}\in\mathcal{C}_{f}^{\beta{(\vartheta })}$ for each fixed $m,n\in\mathbb{N}$ and

$$\begin{aligned} \sup_{m,n\in\mathbb{N}}\sum_{k,l} \Biggl\vert \sum_{j,i=k,l}^{m,n} \biggl(\frac{-s}{r} \biggr)^{j-k} \biggl(\frac{-u}{t} \biggr)^{i-l} \frac {a_{ji}}{rt} \Biggr\vert < \infty. \end{aligned}$$

This means that the *γ*-dual of the space $B(\mathcal{C}_{f})$ is the set $d_{1}\cup CS_{\vartheta}$ as mentioned. □

## Matrix transformations related to the sequence space $B(\mathcal{C}_{f})$

In this section, we characterize some new four-dimensional matrix classes $(B(\mathcal{M}_{u}):\mathcal{C}_{f})$, $(\mathcal {M}_{u}:B(\mathcal{C}_{f}))$. Then we complete this section with some significant results of four-dimensional matrix mapping via the dual summability methods for double sequences which have been introduced and studied by Başar [22] and Yeşilkayagil and Başar [23], and which have recently been applied in [2].

### Theorem 5.1

*A four*-*dimensional matrix* $A=(a_{mnkl})\in(B(\mathcal{C}_{f}):\mathcal {M}_{u})$ *if and only if* $A_{mn}\in\{B(\mathcal{C}_{f})\}^{\beta (\vartheta)}$ *and the following condition holds*:

5.1 $$\begin{aligned} \sup_{m,n\in\mathbb{N}}\sum_{k,l} \Biggl\vert \sum_{i,j=k,l}^{m,n} \biggl( \frac{-s}{r} \biggr)^{i-k} \biggl(\frac{-u}{t} \biggr)^{j-l}\frac {a_{mnij}}{rt} \Biggr\vert < \infty. \end{aligned}$$

### Proof

Suppose that $A=(a_{mnkl})\in(B(\mathcal{C}_{f}):\mathcal{M}_{u})$. Then *Ax* exists and is in $\mathcal{M}_{u}$ for all $x=(x_{mn})\in B(\mathcal{C}_{f})$, which implies that $A_{mn}\in\{B(\mathcal{C}_{f})\} ^{\beta(\vartheta)}$ for all $m,n\in\mathbb{N}$. Thus, we may have the following equality derived from the partial sum of the series $\sum_{k,l}a_{mnkl}x_{kl}$:

5.2 $$\begin{aligned} \sum_{k,l=0}^{m,n}a_{mnkl}x_{kl}&= \sum_{k,l=0}^{m,n}a_{mnkl}\sum _{j,i=0}^{k,l} \biggl(\frac{-s}{r} \biggr)^{k-j} \biggl(\frac{-u}{t} \biggr)^{l-i} \frac{y_{ji}}{rt} \\ &=\sum_{k,l=0}^{m,n}\sum _{j,i=k,l}^{m,n} \biggl(\frac{-s}{r} \biggr)^{j-k} \biggl(\frac{-u}{t} \biggr)^{i-l} \frac{a_{mnji}}{rt}y_{kl} \\ &=(Ey)_{mn}, \end{aligned}$$

where the four-dimensional matrix $E=(e_{mnkl})$ is defined by

$$\begin{aligned} e_{mnkl}=\textstyle\begin{cases} \sum_{j,i=k,l}^{m,n} (\frac{-s}{r} )^{j-k} (\frac {-u}{t} )^{i-l}\frac{a_{mnji}}{rt}& 0\leq k\leq m, 0\leq l\leq n; \\ 0& \text{otherwise,} \end{cases}\displaystyle \end{aligned}$$

for all $m,n\in\mathbb{N}$. Then, by taking the *ϑ*-limit on (5.2) as $m,n\to\infty$, we may say that $Ax=Ey$. Hence, $Ey\in\mathcal{M}_{u}$ whenever $y\in\mathcal{C}_{f}$, that is, $E\in (\mathcal{C}_{f}:\mathcal{M}_{u})$. In this instance, the conditions of Theorem 4.10 hold with $E=(e_{mnkl})$ instead of $A=(a_{mnkl})$, i.e., $E_{mn}\in\{\mathcal{C}_{f}\}^{\beta(\vartheta )}$ and $\sup_{m,n\in\mathbb{N}}\sum_{k,l} \vert e_{mnkl} \vert <\infty$. This completes the proof. □

### Theorem 5.2

*A four*-*dimensional matrix* $A=(a_{mnkl})\in(\mathcal{C}_{f}:B(\mathcal {M}_{u}))$ *if and only if* $A_{mn}\in\{\mathcal{C}_{f}\}^{\beta(\vartheta )}$ *and the following condition hold*:

5.3 $$\begin{aligned} \sup_{m,n\in\mathbb{N}}\sum_{k,l} \Biggl\vert \sum_{i,j=0}^{m,n}b_{mnij}(r,s,t,u)a_{ijkl} \Biggr\vert < \infty. \end{aligned}$$

### Proof

The proof can be shown by the same method as is followed in Theorem 5.1 by using equation (4.6) [24, Theorem 4.7] between the elements of the four-dimensional matrices $A=(a_{mnkl})$ and $G=(g_{mnkl})$. So we omit the details. □

Tuǧ [2] has recently applied the dual summability methods for double sequences which has been introduced and studied by Başar [22], and Yeşilkayagil and Başar [23]. In this work, we use the relation between the four-dimensional matrices $E=(e_{mnkl})$, $e(m,n)$, $G=(g_{mnkl})$ and $H=(h_{mnkl})$ with $A=(a_{mnkl})$, which has been proved and studied in [2, Lemma 4.2, Theorem 4.5].

Now, we may give the relation between the four-dimensional matrices $E=(e_{mnkl})$, $e(m,n)$, $G=(g_{mnkl})$ and $H=(h_{mnkl})$ by

$$\begin{aligned} &e_{mnkl}=\sum_{i,j=k,l}^{m,n} \biggl( \frac{-s}{r} \biggr)^{i-k} \biggl(\frac{-u}{t} \biggr)^{j-l}\frac{a_{mnij}}{rt}, \\ &e(m,n)=\sum_{k,l=0}^{m,n}\sum _{i,j=k,l}^{\infty} \biggl(\frac{-s}{r} \biggr)^{i-k} \biggl(\frac{-u}{t} \biggr)^{j-l} \frac{a_{mnij}}{rt}, \\ &g_{mnkl}=\sum_{i,j=0}^{m,n}b_{mnij}a_{ijkl}, \quad\text{and} \\ &h_{mnkl}=\sum_{i,j=k,l}^{m,n}b_{mnij}e_{ijkl} \end{aligned}$$

for all $m,n,k,l\in\mathbb{N}$.

Now we may give the following new significant results for the four-dimensional infinite matrix $A=(a_{mnkl})$.

### Corollary 5.3

*The following statements hold*.

(i) $A\in(B(\mathcal{C}_{bp}):\mathcal{C}_{f})$ *iff* (4.1)-(4.5) *hold with* $e_{mnkl}$ *instead of* $a_{mnkl}$.
(ii) $A\in(B(\mathcal{C}_{r}):\mathcal{C}_{f})$ *iff* (4.1)-(4.3) *and* (4.6)-(4.7) *hold with* $e_{mnkl}$ *instead of* $a_{mnkl}$.
(iii) $A\in(B(\mathcal{C}_{p}):\mathcal{C}_{f})$ *iff* (4.1)-(4.3) *hold with* $e_{mnkl}$ *instead of* $a_{mnkl}$.
(iv) $A\in(B(\mathcal{M}_{u}):\mathcal{C}_{f})$ *iff* (4.1) *and* (4.14)-(4.16) *hold with* $e_{mnkl}$ *instead of* $a_{mnkl}$.
(v) $A\in(B(\mathcal{C}_{f}):\mathcal{C}_{bp})$ *iff* (4.1) *and* (4.8)-(4.13) *hold with* $e_{mnkl}$ *instead of* $a_{mnkl}$.

### Corollary 5.4

*The following statements hold*.

(i) $A\in(\mathcal{C}_{p}:B(\mathcal{C}_{f}))$ *iff* (4.1)-(4.3) *hold with* $g_{mnkl}$ *instead of* $a_{mnkl}$.
(ii) $A\in(\mathcal{C}_{bp}:B(\mathcal{C}_{f}))$ *iff* (4.1)-(4.5) *hold with* $g_{mnkl}$ *instead of* $a_{mnkl}$.
(iii) $A\in(\mathcal{C}_{r}:B(\mathcal{C}_{f}))$ *iff* (4.1)-(4.3) *and* (4.6)-(4.7) *hold with* $g_{mnkl}$ *instead of* $a_{mnkl}$.
(iv) $A\in(\mathcal{M}_{u}:B(\mathcal{C}_{f}))$ *iff* (4.1) *and* (4.14)-(4.16) *hold with* $g_{mnkl}$ *instead of* $a_{mnkl}$.
(v) $A\in(\mathcal{C}_{f}:B(\mathcal{C}_{bp}))$ *iff* (4.1) *and* (4.8)-(4.13) *hold with* $g_{mnkl}$ *instead of* $a_{mnkl}$.

### Corollary 5.5

*The following statements hold*.

(i) $A\in(B(\mathcal{C}_{p}):B(\mathcal{C}_{f}))$ *iff* (4.1)-(4.3) *hold with* $h_{mnkl}$ *instead of* $a_{mnkl}$.
(ii) $A\in(B(\mathcal{C}_{bp}):B(\mathcal{C}_{f}))$ *iff* (4.1)-(4.5) *hold with* $h_{mnkl}$ *instead of* $a_{mnkl}$.
(iii) $A\in(B(\mathcal{C}_{r}):B(\mathcal{C}_{f}))$ *iff* 4.1)-(4.3) *and* (4.6)-(4.7) *hold with* $h_{mnkl}$ *instead of* $a_{mnkl}$.
(iv) $A\in(B(\mathcal{M}_{u}):B(\mathcal{C}_{f}))$ *iff* (4.1) *and* (4.14)-(4.16) *hold with* $h_{mnkl}$ *instead of* $a_{mnkl}$.
(v) $A\in(B(\mathcal{C}_{f}):B(\mathcal{C}_{bp}))$ *iff* (4.1) *and* (4.8)-(4.13) *hold with* $h_{mnkl}$ *instead of* $a_{mnkl}$.
(vi) $A\in(B(\mathcal{C}_{f}):B(\mathcal{M}_{u}))$ *iff* (4.1) *hold with* $h_{mnkl}$ *instead of* $a_{mnkl}$.

### Corollary 5.6

*The following statements hold*.

(i) $A\in(B(\mathcal{C}_{f}):\mathcal{CS}_{bp})$ *iff* (4.1) *and* (4.8)-(4.13) *hold with* $e(m,n)$ *instead of* $a_{mnkl}$.
(ii) $A\in(B(\mathcal{C}_{f}):\mathcal{BS})$ *iff* (4.1) *hold with* $e(m,n)$ *instead of* $a_{mnkl}$.

### Corollary 5.7

*The following statements hold*.

(i) $A\in(B(\mathcal{C}_{f}):\mathcal{C}_{f};p)$ *iff* (4.1), (4.17)-(4.22) *hold with* $e_{mnkl}$ *instead of* $a_{mnkl}$.
(ii) $A\in(\mathcal{C}_{f}:B(\mathcal{C}_{f});p)$ *iff* (4.1), (4.17)-(4.22) *hold with* $g_{mnkl}$ *instead of* $a_{mnkl}$.
(iii) $A\in(B(\mathcal{C}_{f}):B(\mathcal{C}_{f});p)$ *iff* (4.1), (4.17)-(4.22) *hold with* $h_{mnkl}$ *instead of* $a_{mnkl}$.

## Conclusion

The concept of almost convergence of single sequence was introduced by Lorentz [12]. In 2010, Mursaleen [25] investigated the certain properties of the space of almost convergent sequences denoted by *f*. Then many mathematicians have studied the matrix domain on almost null and almost convergent sequences spaces (see [26–29]).

The almost convergence for double sequence was introduced by Moricz and Rhoades [13] and studied by many researchers (see [15, 21, 30–38]). Yeşilkayagil and Başar [19] recently studied the topological properties of the spaces of almost null and almost convergent double sequences.

In this work, we studied the domain of the four-dimensional generalized difference matrix $B=(b_{mnkl})$ in the spaces of almost null and almost convergent double sequences and examined some topological properties. Moreover, we determined the *α*-, $\beta(bp)$- and *γ*-duals of the space $B(\mathcal{C}_{f})$ and characterized some new classes of four-dimensional matrix mappings related with the sequence space $B(\mathcal{C}_{f})$. The characterization of the matrix classes $(\mathcal{C}_{f}: \mathcal{C}_{p})$, $(\mathcal {C}_{f}: \mathcal{C}_{r})$, $(B(\mathcal{C}_{f}): \mathcal{C}_{p})$ and $(B(\mathcal{C}_{f}): \mathcal{C}_{r})$, and $(\mathcal {L}_{s'}:\mathcal{C}_{f})$, $(B(\mathcal{L}_{s'}):\mathcal{C}_{f})$, $(\mathcal{L}_{s'}:B(\mathcal{C}_{f}))$ in the two cases $0< s'<1$ and $1< s'<\infty$, and the $\beta(p)$-dual and $\beta(r)$-dual of the space $B(\mathcal{C}_{f})$ are still open problems.
